# Long QT Syndrome Type 2: Emerging Strategies for Correcting Class 2 *KCNH2* (*hERG*) Mutations and Identifying New Patients

**DOI:** 10.3390/biom10081144

**Published:** 2020-08-04

**Authors:** Makoto Ono, Don E. Burgess, Elizabeth A. Schroder, Claude S. Elayi, Corey L. Anderson, Craig T. January, Bin Sun, Kalyan Immadisetty, Peter M. Kekenes-Huskey, Brian P. Delisle

**Affiliations:** 1 Department of Physiology, Cardiovascular Research Center, Center for Muscle Biology, University of Kentucky, Lexington, KY 40536, USA; makoto.ono@uky.edu (M.O.); deburgess@uky.edu (D.E.B.); eschr0@uky.edu (E.A.S.); 2 CHI Saint Joseph Hospital, Lexington, KY 40504, USA; samyelayi@sjhlex.org; 3 Cellular and Molecular Arrhythmia Research Program, University of Wisconsin, Madison, WI 53706, USA; clanders@medicine.wisc.edu (C.L.A.); ctj@medicine.wisc.edu (C.T.J.); 4 Department of Cellular & Molecular Physiology, Loyola University Chicago, Chicago, IL 60153, USA; bsun@luc.edu (B.S.); kimmadisetty@luc.edu (K.I.); pkekeneshuskey@luc.edu (P.M.K.-H.)

**Keywords:** long QT syndrome, ion channel, trafficking, *KCNH2*, *hERG*

## Abstract

Significant advances in our understanding of the molecular mechanisms that cause congenital long QT syndrome (LQTS) have been made. A wide variety of experimental approaches, including heterologous expression of mutant ion channel proteins and the use of inducible pluripotent stem cell-derived cardiomyocytes (iPSC-CMs) from LQTS patients offer insights into etiology and new therapeutic strategies. This review briefly discusses the major molecular mechanisms underlying LQTS type 2 (LQT2), which is caused by loss-of-function (LOF) mutations in the *KCNH2* gene (also known as the human ether-à-go-go-related gene or *hERG*). Almost half of suspected LQT2-causing mutations are missense mutations, and functional studies suggest that about 90% of these mutations disrupt the intracellular transport, or trafficking, of the *KCNH2*-encoded Kv11.1 channel protein to the cell surface membrane. In this review, we discuss emerging strategies that improve the trafficking and functional expression of trafficking-deficient LQT2 Kv11.1 channel proteins to the cell surface membrane and how new insights into the structure of the Kv11.1 channel protein will lead to computational approaches that identify which *KCNH2* missense variants confer a high-risk for LQT2.

## 1. Introduction

Congenital long QT syndrome (LQTS) is an arrhythmogenic disorder that can manifest as an abnormal prolongation in the heart rate-corrected QT (QTc) interval measured on an electrocardiogram (ECG) [1,2]. It delays ventricular repolarization and increases the probability of life-threatening polymorphic ventricular tachycardia called torsades de pointes (TdP) [3]. LQTS has been identified all over the world with a prevalence as high as 1 in 2500 healthy live births [4]. The major autosomal dominant causes of LQTS are linked to rare mutations in one of three cardiac ion channel genes: *KCNQ1* (LQT1), *KCNH2* (LQT2), and *SCN5A* (LQT3) [5,6,7,8,9,10,11]. Most patients with LQTS can be successfully treated with drugs that block β-adrenergic receptors (β-blockers), life-style interventions (e.g., avoid QT prolonging drugs, hypokalemia and hypomagnesemia), and implantable cardioverter defibrillators (ICDs) for high-risk clinical features [12,13,14].

### 1.1. KCNH2, Kv11.1 and I_Kr_

**Figure 1:**
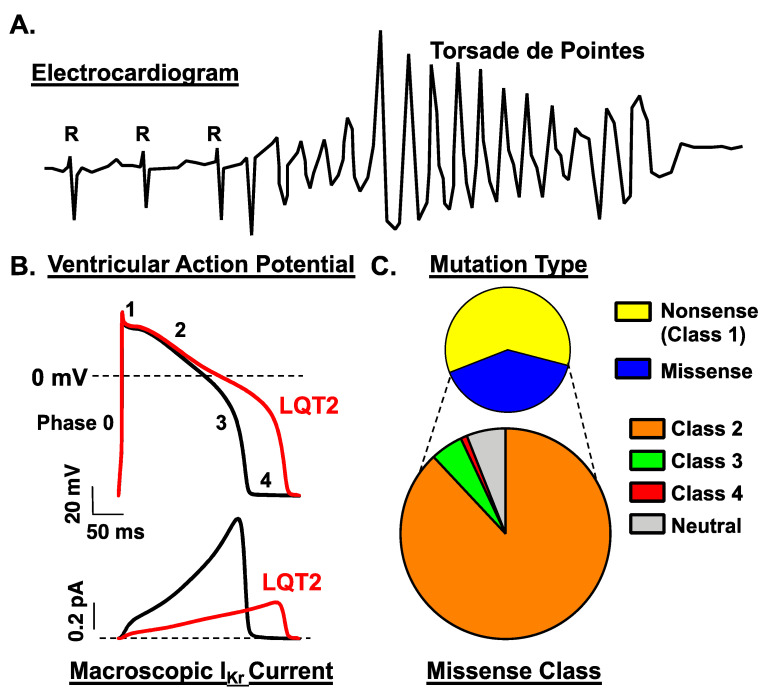
(**A**). This cartoon of an electrocardiogram recording shows normal sinus rhythm with a long QT interval that degenerates into Torsade de Pointes. (**B**). Loss-of-function mutations in *KCNH2* that decrease the amplitude of I_Kr_ increase the ventricular action potential duration. The top panel shows a ventricular action potential with the different phases of the action potential labeled (0, 1, 2, 3, and 4) in control conditions (black traces) or after a loss in I_Kr_ (LQT2, red). The bottom panel shows the corresponding I_Kr_. The dashed lines represent 0 mV or 0 pA. (**C**). The top pie chart shows the % of candidate LQT2 mutations that are nonsense (Class 1) or missense. To generate this graph, we defined candidate LQT2 missense mutations as ones that are reported to be “likely pathogenic” or “pathogenic” in ClinVar (https://www.ncbi.nlm.nih.gov/clinvar). The bottom pie chart shows the relative percentage of LQT2 missense mutations that are expected to cause LQTS by a Class 2, 3, or 4 mechanism. These data are based on the functional studies of missense variants reported by Anderson and colleagues (2006 and 2014) [24,25]. A few mutations did not have an obvious dysfunctional phenotype (neutral) and might represent *KCNH2* VUS misclassified as causing LQT2.

Loss-of-function (LOF) mutations in *KCNH2* are a leading cause of LQTS [15]. *KCNH2* encodes the RNA that is translated into voltage-gated K^+^ channel Kv11.1 *α*-subunit channel proteins [5,6]. These α-subunit channel proteins form channels that conduct the rapidly activating delayed rectifier K^+^ current (I_Kr_) in the cardiomyocyte sarcolemma membrane. A loss of I_Kr_ can delay the ventricular AP repolarization to cause LQTS (Figure 1). The channels that conduct I_Kr_ in cardiomyocytes are minimally generated by the *KCNH2* gene products Kv11.1a and Kv11.1b α-subunits and the auxiliary subunit MiRP1 (*KCNE2*) [16,17,18,19]. Kv11.1a is the full-length, 15-exon, 1159 amino acid *α*-subunit (*hERG1a* or *KCNH2a*), and Kv11.1b is generated from an alternate start site and 5′ exon that replaces the full-length 1–5 exons (*hERG1b* or *KCNH2b*) [17]. Alternative splicing and polyadenylation of pre-mRNA generate truncated versions of Kv11.1a and Kv11.1b transcripts (Kv11.1a-USO or Kv11.1b-USO), but these gene products are not functional [20,21]. MiRP1 associates with Kv11.1 channel proteins and several other ion channels in the heart [22]. MiRP mutations were previously thought to cause autosomal dominant LQTS, but recent clinical data has invalidated most if not all of these variants [23]. Since the importance of MiRP in LQTS remains unclear, this review focus on the studies that investigate the cellular and molecular mechanisms that cause LOF for LQT2 mutations in Kv11.1a channels.

**Figure 2:**
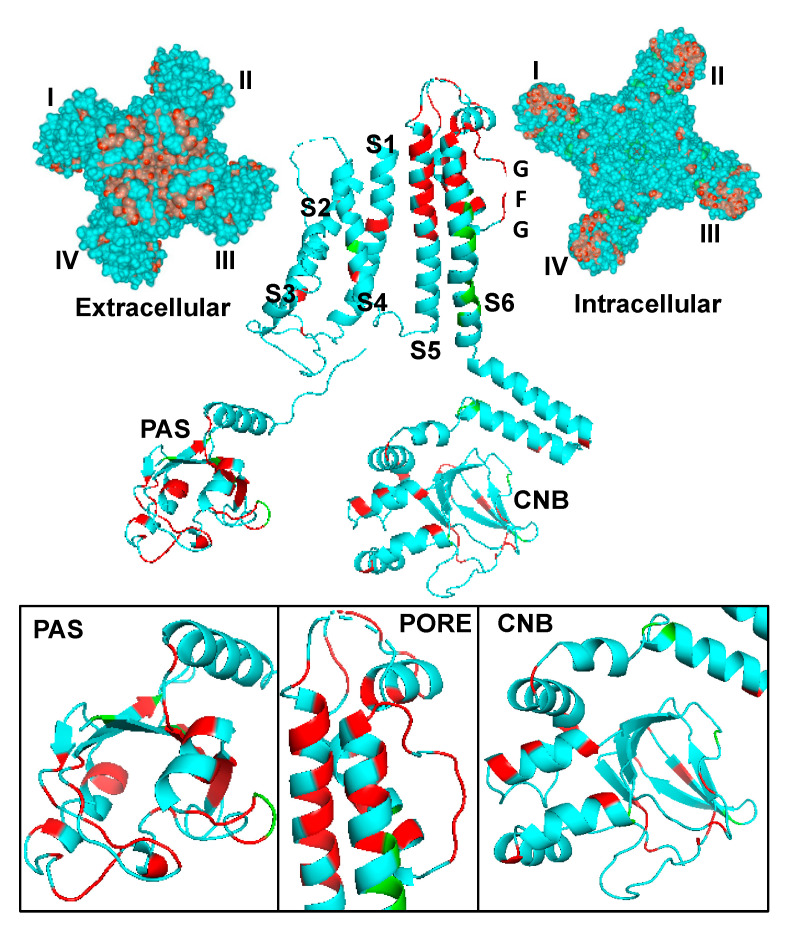
A CryoEM structure of Kv11.1a channel as viewed from the extracellular and intracellular side of the membrane (top left and right, each Kv11.1a α-subunit is denoted I-IV). A side-view of an individual Kv11.1a α-subunit highlighting the selectivity filter (G-F-G) and the secondary structures of the PAS, PORE, and CNB domain (middle and bottom). The location of LQT2 missense mutations that have been tested and disrupt Kv11.1a channel trafficking is shown in red. Green denotes the location of LQT2 missense mutations that have been tested and do not disrupt Kv11.1a trafficking.

Kv11.1 channel α-subunits contain cytosolic amino (NH_2_) and carboxyl (COOH) termini and six α-helical transmembrane segments (S1–S6) [26,27]. Atom density maps of detergent/lipid-solubilized Kv11.1a channel proteins show that the voltage sensor domain is formed by S1–S4 and the pore domain is formed by S5 and S6 (Figure 2) [27]. The proposed sequence of events for Kv channel biogenesis is: translation and insertion in the endoplasmic reticulum (ER) membrane, the addition of asparagine-linked (N-linked) glycans, the tetramerization of the *α*-subunits, and the correct or native folding of the voltage-sensor, pore, NH_2_ and COOH termini [28].

The ER extends from the cell nucleus out to the cell periphery [29]. It has several compartments that participate in the biosynthesis of luminal, secretory, membrane associated, and integral membrane proteins and lipids; Ca^2+^ storage and release; apoptotic signaling; quality control; ER-associated degradation; and ER export. The environment in the ER optimizes newly synthesized protein folding and quality control mechanisms prevent the trafficking of incompletely assembled or misfolded proteins to their functional destination in the cell. The folding of newly synthesized proteins and ER associated degradation of misfolded proteins is regulated by molecular chaperone proteins.

**Figure 3:**
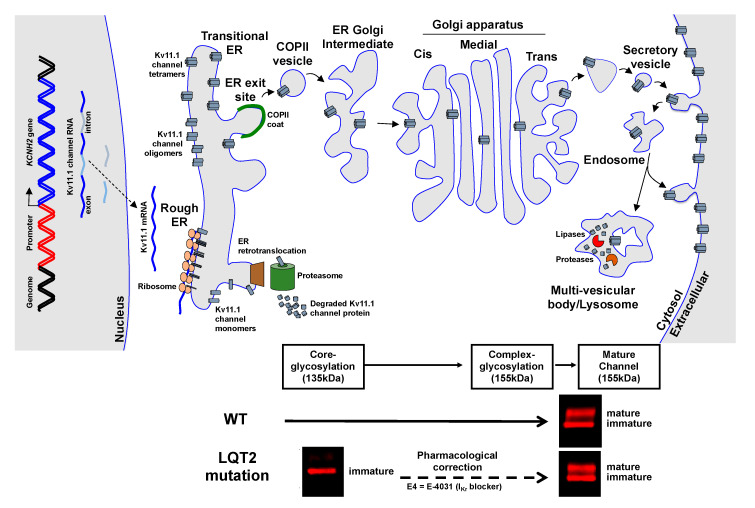
A cartoon diagram of the Kv11.1 channel trafficking pathway. *KCNH2* is transcribed and spliced to mRNA, which is then translated in the ER as a core glycosylated Kv11.1a *α*-subunit with a molecular mass of 135 kDa. It undergoes terminal glycosylation as it traffics through the Golgi apparatus and its molecular weight increases to 155 kDa. Kv11.1 channels recycle on and off the membrane in endosomes every few minutes for several hours before being degraded in the lysosome pathway. Representative Western blots of cells expressing WT-Kv11.1a *α*-subunit or a Class 2 LQT2 channel protein are shown below the cartoon. In control conditions, the immunoblot of cells expressing WT-Kv11.1a contains both core and terminally glycosylated Kv11.1a protein bands, whereas cells expressing the Class 2 LQT2 mutant channel protein show only the core glycosylated form. Incubating cells in I_Kr_ blockers (e.g., E-4031) increases terminal glycosylation and functional expression of Class 2 LQT2 channel proteins (pharmacological correction, dashed arrow).

Kv11.1a α-subunit channel proteins are synthesized in the ER as an N-linked glycoproteins with a molecular mass of ~135 kDa in the ER (Figure 3) [30]. Chaperone proteins in the ER lumen and the cytosol regulate the folding and ER associated degradation of misfolded Kv11.1 channel proteins [31,32]. Once the Kv11.1 channel proteins achieve their native conformation, they are exported out of the ER into the secretory pathway in coatomer associated protein II (COPII) vesicles to the ER Golgi intermediate compartment (ERGIC) and Golgi compartment [33]. As Kv11.1a proteins traffic through the Golgi apparatus, the N-linked glycans on Kv11.1 α-subunit channel proteins are terminally glycosylated and their molecular mass increases to ~155 kDa [34]. At the cell surface membrane, Kv11.1 channels are continually internalized and recycle back to the cell surface membrane every couple of minutes for several hours before they are targeted for degradation in lysosomes [35,36] There are several excellent reviews that detail Kv11.1 channel trafficking, chaperones, and the biophysical function of Kv11.1 channels/I_Kr_ [15,16,31,37] This review is focused on the newer strategies being developed to treat LQT2 and identify new LQT2 patients before they suffer a life-threatening event.

### 1.2. Long QT Syndrome Type 2

LQT2 mutations are classified by the molecular mechanism with which they decrease I_Kr_ [15]. Class 1 mutations disrupt the synthesis of the *KCNH2*-encoded Kv11.1 channel subunits; Class 2 mutations disrupt the intracellular transport or trafficking of Kv11.1 channel proteins to the cell membrane; Class 3 mutations disrupt I_Kr_ channel gating; and Class 4 mutations disrupt I_Kr_ channel permeation/selectivity. Most LQT2-linked mutations decrease Kv11.1 channel number in the cell surface membrane by a Class 1 or 2 mechanism. A little more than half of LQT2-linked mutations are nonsense and most predict haploinsufficiency via nonsense mediated RNA decay (NMD) (Class 1 mechanism) [38,39,40]. The remaining LQT2-linked mutations are rare missense mutations, and heterologous expression studies show that about 90% of suspected LQT2 missense mutations disrupt the trafficking of the full length Kv11.1a channel proteins to the cell surface membrane (Class 2 mechanism) (Figure 1) [24,25,41]. These data suggest that the majority of LQT2 cases are linked to mutations that result in a decrease in maximal current. However, the identification of several Class 3 mutations suggests that disruptions in Kv11.1a channel gating also result in LQT2 [42]. Class 3 mutations are expected to reduce the open probability during the repolarization phase of the ventricular AP.

Most Class 2 LQT2 mutations have been identified using Western blot of cells heterologously expressing mutant Kv11.1a channel proteins. Cells expressing Class 2 LQT2 Kv11.1a channel proteins do not generate a 155 kDa Kv11.1a protein band (Figure 3). Some Class 2 Kv11.1a channel proteins generate tetramers that are sequestered throughout the transitional ER compartment, whereas others disrupt the co-assembly of mutant Kv11.1a α-subunits and are targeted for ER associated degradation [32,43,44,45,46,47,48,49,50]. Class 2 Kv11.1a channel proteins that generate tetramers might cause dominant negative (DN) effects by co-assembling and inhibiting the trafficking of WT-Kv11.1a channel proteins [51]. In contrast, Class 2 Kv11.1a mutations that disrupt co-assembly might generate haploinsufficient molecular phenotypes, whereby only the mutant Kv11.1a channel proteins are affected.

### 1.3. Novel Therapeutic Approaches to Treat LQT2

β-blocker medications and cardiac sympathetic denervation prevent life threatening ventricular arrhythmias, whereas implantable cardioverter defibrillators (ICDs) can terminate these arrhythmias. However, some patients are contraindicated or refractory to β-blocker and ICDs or cardiac sympathetic denervation can result in significant surgical complications [52]. Therefore, researchers are continually working to identify alternative and more effective pharmacological strategies to treat patients with LQT2.

A challenge to treating patients with Class 2 LQT2 mutations is that there is no one dominant disease-causing mutation, but rather hundreds of different Class 2 LQT2 missense mutations that span the Kv11.1a *α*-subunit (Figure 2) [25]. It is increasingly clear that the identity and location of LQT2-mutations in the Kv11.1a *α-subunit* are critical determinants for Kv11.1 channel protein trafficking [24]. Most Class 2 LQT2 mutations localize to three major structural domains in the Kv11.1a α-subunit: N-terminal Per-Arnt-Sim domain (PASD), the pore domain, or the C-terminal cyclic nucleotide-binding domain (CNBD). These trends were largely identified through mapping variants identified from genetic screens to the primary sequences [24].

A surprising finding is that incubating cells with drugs that bind to Kv11.1 channel proteins and block I_Kr_ (I_Kr_ blockers) can increase the trafficking for about one third of Class 2 LQT2 mutations (Figure 3) [24,25,53]. Electrophysiological experiments show that pharmacological correction of Class 2 LQT2 Kv11.1a channel protein trafficking also increases functional expression of the mutant channel proteins after drug wash out. A comprehensive analysis of LQT2-linked missense mutations in Kv11.1a channel proteins demonstrated a relationship between their location and ability to undergo pharmacological correction with I_Kr_ blockers [25]. Western blot analysis for over 100 mutant Class 2 LQT2 channel proteins showed that culturing cells in the high-affinity I_Kr_ blocker E-4031 increased the terminal glycosylation for 47% of Kv11.1a channel proteins with Class 2 mutations in the PAS domain, 33% of the Kv11.1 channel proteins with Class 2 mutations in the pore domain, and 21% of the Kv11.1 channel proteins with Class 2 mutations in the CNB domain. Unfortunately, these drugs have limited therapeutic potential since they block I_Kr_ and cause drug-induced LQTS.

Most drugs that block I_Kr_ bind to the inner aqueous vestibule at the Y652 and F656 residue in S6 of the Kv11.1a α-subunit [54,55]. Ficker and colleagues (2002) found that engineering amino acid substitutions at F656 reduced both the affinity of the drug blocker of I_Kr_ and the pharmacological correction of Class 2 LQT2 channel proteins [45]. Conversely, engineering certain amino acid substitutions at the Y652 residue (e.g., Y652C) mitigated the trafficking-deficient phenotypes for several different Class 2 LQT2 mutations [56]. These latter data are an example of intragenic suppression, whereby variants at Y652 compensate for the primary misfolding defect caused by disease-causing Class 2 LQT2 mutation. Taken together, the results demonstrate that cleanly separating drug block and pharmacological correction might not be possible.

I_Kr_ activators increase the open probability of Kv11.1a channels at the cell surface membrane and might be used as pharmacotherapy for LQT2 [57]. However, they could lead to abnormal shortening of the QT interval and increase the risk for deadly arrhythmias like ventricular fibrillation. Qile and colleagues (2020) determined whether the functional expression of Class 2 LQT2 Kv11.1 channels could be increased by culturing cells in I_Kr_ blockers and the I_Kr_ activator LUF7244 [58,59]. LUF7244 is an allosteric I_Kr_ activator that counteracts I_Kr_ blocker induced arrhythmias in dogs. Heterologous expression studies showed that this strategy of combining drugs that increase trafficking and open probability of Kv11.1a channels was effective at increasing the functional expression of Class 2 LQT2 channel proteins at the cell surface membrane (even without drug wash out). This raises the possibility that I_Kr_ blockers and I_Kr_ activators could be used together to increase mutant channel trafficking and function at the cell surface membrane.

Many different congenital diseases are caused by mutations that disrupt protein folding and trafficking [60]. One of the best studied diseases caused by mutations that disrupt ion channel trafficking is cystic fibrosis [61,62,63]. Cystic fibrosis is caused by LOF mutations in the *cystic fibrosis transmembrane conductance regulator* (*CFTR*). *CFTR* encodes an ABC transporter-class Cl^−^ channel and most cases of cystic fibrosis cases are linked to the deletion mutation (F508del-CFTR), which increases CFTR channel protein misfolding and decreases its trafficking out of the ER [64,65]. Recent investigational therapeutic strategies to treat CFTR include the drugs that can facilitate the native folding and membrane expression of CFTR channels (lumacaftor), as well as drugs that increase the open probability of CFTR channels (ivacaftor) [66,67].

Mehta and colleagues (2018) determined whether lumacaftor also improves the trafficking of Class 2 LQT2 channel proteins [68]. These studies began with in vitro testing in inducible pluripotent stem cell-derived cardiomyocytes (iPSC-CMs) generated from patients that have Class 2 LQT2 mutations. Incubating iPSC-CMs in lumacaftor shortened the field potential duration, which is the iPSC-CM rough equivalent to the QT interval [68]. Schwartz and colleagues [69] tested whether lumacaftor and ivacaftor could normalize the QT interval in several LQT2 patients who harbor the same Class 2 LQT2 mutation as in iPSC-CMs studies. The patients treated with these drugs showed shortening in their QTc interval, but the absolute magnitude was less than expected based on the iPSC-CM data and the patients suffered some undesirable side effects. This discrepancy could be caused by differences to drug delivery to iPSC-CMs in a dish vs. an intact organ, the phenotypic immaturity of iPSC-CMs, and/or the observation that iPSC-CMs express a relatively large I_Kr_ [70]. Additional considerations could be the systemic changes that these drugs might cause on physiological variables important for maintaining the QT interval. The authors concluded that the use of this drug combination for treating LQT2 patients is premature until larger patient populations can be studied.

Although improving the trafficking of Class 2 Kv11.1a channel proteins represents an attractive therapeutic strategy, it might be impractical because of the large number of different mutations and their diverse trafficking and biophysical molecular phenotypes (for review see [71]). It is clear that pharmacological correction strategies improve the trafficking of certain mutations but not others. Even if pharmacological correction of Class 2 Kv11.1a channel proteins can be done in patients, there is no guarantee that the corrected mutant channel proteins will function normally to mitigate the clinical phenotype. Indeed, increasing the trafficking of mutant channel proteins that also severely disrupt Kv11.1a channel gating and/or permeation could worsen the disease.

### 1.4. KCNH2 Variants of Uncertain Significance (VUS)

Since we already have several effective therapies to currently treat patients with LQT2, then genetic screening of large patient populations could facilitate the early identification and prophylactic treatment [72,73]. A challenge is that most genetic tests and Whole Genome/Exome Sequencing identify novel, rare *KCNH2* missense variants of uncertain physiological significance (VUS). The vast majority of these VUS are likely neutral because the allelic frequencies for a VUS in *KCNH2* far outpaces the true incidence of the disease [12,74,75,76,77]. Therefore, positive genetic tests for VUS are not actionable.

One might think that laboratories experienced in genetic testing, ion channels, and cardiac arrhythmias would be good at predicting which LQT2-linked VUS are potentially pathogenic. However, this is proving to be extremely difficult. Van Driest et al. (2016) [78] found that, not only was there a discordance between different laboratories in designating a LQTS-linked VUS as pathogenic, but also that certain VUS identified as pathogenic did not associate with autosomal dominant LQTS. An incomplete understanding in the probabilistic nature of genetic testing, coupled with unexpected high allelic frequencies of neutral VUS, has already contributed to LQTS misdiagnoses and unnecessary treatment in patients, including invasive procedures with potential complications [52,79]. The current clinical recommendations now limit genetic screening to phenotypically positive LQT2 patients to prevent the misdiagnosis and over treatment of patients [12,80]. Since one of the presenting symptoms of LQT2 is sudden cardiac death, patients would benefit from the development of reliable gene-first strategies that identify LOF *KCNH2* VUS before they experience a life-threatening arrhythmia.

**Figure 4:**
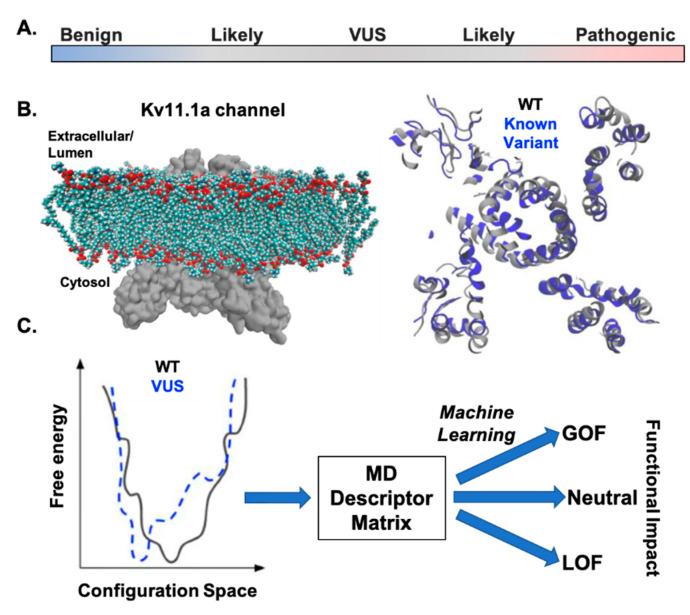
(**A**). The American College of Medical Genetics and Genomics and the Association of Molecular Pathology provides a rubric that can be used to classify variants identified in Mendelian-linked disease alleles as pathogenic, likely pathogenic, uncertain significance, likely benign, and benign. (**B**). Shown is an example of how molecular dynamics (MD) simulations could be used to determine the structural impactof known and newly identified variants on Kv11.1a channel configurations. (**C**). MD simulations could be used to relate structural configurations to thermodynamic changes, like those controlling folding, that cause a LOF, gain of function (GOF) or neutral phenotype. Such predictions could be compiled for known variants into a descriptor matrix to train machine learning approaches for de novo prediction of the structural and functional impact of newly identified VUS in Kv11.1a channels. Over time, the sensitivity of these approaches might be fine-tuned to determine which VUS act as genetic modifiers.

A joint consensus guideline document from the American College of Medical Genetics and Genomics and the Association of Molecular Pathology provides some guidance for the interpretation of sequence variants [81]. The rubric can be used to classify variants identified in Mendelian-linked disease alleles as pathogenic, likely pathogenic, uncertain significance, likely benign, and benign (Figure 4A). The rubric to classify variants uses population data (mean allelic frequency), computational data, functional data, and segregation data. It can be useful, but without functional data, it does not inform how predicted pathogenic or likely pathogenic mutations affect function. Additionally, it does not mention the possibility of identifying genetic variants that act as genetic modifiers. These are *KCNH2* variants that do not cause autosomal dominant LQT2 but rather modify the risk of having LQTS in response to disease, drugs, or the presence of genetic variants in other LQTS-susceptibility genes. We expect that advances in computational and structural biology will help not only determine how specific variants impact the structure but also the function for a protein of interest (Figure 4B,C). Ideally, computer simulations of known variants in Kv11.1a protein structures can be used to identify structural features toward predicting which newly identified VUS are neutral, genetic modifiers, or result in a LOF (or gain of function) by a Class 2, 3, or 4 mechanism.

In recent years, bioinformatics and protein-structure prediction approaches have leveraged primary structural information to infer structural or functional consequences of protein variants [82]. Disappointingly, while the PAS, pore and CNBD appear to harbor the majority of LQT2-linked variants, few correlations between the clinical severity and nature of their loss-of-function defects have emerged. Dozens of missense variants identified within or near these sequences present no obvious functional or clinical phenotype [83,84]. This severely undermines the utility of using sequence position alone as a way to infer LOF Kv11.1a variants.

Extending beyond analyses of the KCNH2 primary sequence alone, Anderson and colleagues provided considerable insights into how mutation-specific differences in the physicochemical properties and location of missense LQT2 variants in the PAS domain impact trafficking [85]. In this study, an in vitro assay reporting on protein solubility demonstrated that Class 2 LQT2 mutations in the PAS domain were considerably less soluble than their WT and trafficking-permissive counterparts. Further, the severity of trafficking defects loosely correlated with their relative insolubility as measured by the assay when compared to the WT Kv11.1a PAS domain. Interestingly, variants that undergo pharmacologic correction with I_Kr_ blockers were generally only mildly insoluble. It is tempting to speculate that mutant Kv11.1a channel proteins that undergo pharmacological correction generate relatively minor structural perturbations.

Ascribing physicochemical properties such as hydrophobicity based on single amino acid substitutions and primary sequence alone to predict the impact of an individual mutation on Kv11.1a function is challenging. Bioinformatics approaches have nonetheless identified statistically significant associations between folding free energies and genetic sequences [86,87]. Differences in Kv11.1a channel folding free energies can serve as suggestive indicators of misfolding and defective trafficking, as shown in Anderson et al. [25] It is important to remember that the effects of missense variants are not restricted to only the regions of the protein that are adjacent in primary sequence, but they can act allosterically, as they couple to and depend on the structural configuration of the entire protein. The interdependence of adjacent and nonadjacent sequence information in determining the impact of a mutation on channel function underscores the need for incorporating structural information for predicting the functional impact of individual variants [88].

Effects of mutations on Kv11.1a protein function can be interpreted on the basis of how they impact secondary (alpha helix, random coil, beta sheets), tertiary (protein and subdomain folding) and quaternary (protein/protein associations) factors. Myriad algorithms have been introduced to predict protein folding tendencies for forming canonical secondary structures, identify protein folds based on sequence similarities to other protein structures deposited in the Protein Databank, and predict potential protein–protein interaction interfaces [82,89,90,91]. Nonetheless, a gold standard for surmising structure/function relationships in proteins and how they might be perturbed by missense mutations is interpreting atomistic-scale structural data of intact proteins, when available. To date, such information for Kv11.1a is incomplete and comprises only Angstrom resolution models of its PAS, EAG and CNBD domains but not of the whole channel [92,93,94]. However, a significant advance in this regard is the recent availability of sub-nanometer resolution structural data of the Kv11.1a channel via cryo-electron microscopy [27]. This structure has been critical in the initial stages of interpreting how known LQT2 mutations, as well as newly identified KCNH2 VUS, impact Kv11.1a properties and its function.

Computational simulation techniques including homology-modeling and molecular dynamics simulations (MDS) are among the most popular tools for predicting the structural bases of protein function and potential changes in function from mutations. Homology models are used to predict the likely three-dimensional structure of a primary sequence using structural data available in homologous proteins as templates [95]. The accuracy of these approaches are highly dependent on the quality of the template data and sequence identity. Molecular simulations generally use physics- or knowledge-based scoring functions to estimate interaction potentials between amino acids or atoms, which are used to predict three-dimensional protein structures or how they move under those potentials [96]. Recently, cryo-electron microscopy based models and MDS simulations of Kv11.1a channel proteins have been used to elucidate a gain-of-function (GOF) mutation that impacts the channel’s selectivity filter [97]. This study was a first-of-its-kind approach for linking KCNH2 genomic information to a functional outcome using structurally-realistic models of the channel and computer simulations.

While the use of molecular simulations of the Kv11.1a channel represent a significant and innovative step toward predicting the pathogenicity of KCNH2 variants, several advancements are still needed to fully realize this potential. Cryo-electron data are relatively low resolution (sub nanometer) compared to crystallography- or NMR-derived structures (Angstrom) limiting the ability to conclusively link a mutation with its mechanism of dysfunction. While a recent advancement in cryo-electron microscopy has resolved individual atoms in apoferritin [98], it is unclear when such resolution gains may be accomplished for membrane channels like Kv11.1a. In the meantime, continued improvements in the solubilization and crystallization of membrane proteins including ion channels will be required to approach atomistic resolution images via X-ray crystallography, as it has been done in recent years with voltage-gated Na^+^ channels [99]. Additionally, efficiently implementation of MDS will benefit from force fields that include the effects of electrostatic polarization as the local electric field changes [100].

MDS based on these structural data, regardless of the structural resolution, remain intractable for modeling molecular events at microsecond or longer timescales relevant for biological function. Recent advances in specialized hardware, including graphics processing units and chip architectures, have begun to approach these timescales and have been used to probe mechanisms of channel folding and function (reviewed in [101]) [102,103]. In this regard, molecular simulations leveraging these technological advancements could enable predictions of potential trafficking behavior from first principles for the vast majority of known mutations and future KCNH2 VUS. To realize this outcome, computer simulations of these processes will require defining and determining the structural regions of the Kv11.1a channel implicated in defective trafficking, such as its PAS domain, as well as highlighting properties like solubility that correlate with LOF. Similarly, the resolution of complexes between the channel and chaperones including heat shock proteins may further yield important mechanistic clues for how missense variants impact ER quality control and trafficking [31,32].

Naturally, a compelling motivation for using computational techniques to determine relationships between genetic information and Kv11.1a dysfunction is to provide predictive information that is superior to genetic screening but not as labor intensive, expensive, or slow as functional studies using in vitro or iPSC-CMs. Achieving this prediction capacity remains the critical barrier to using genetic screening more effectively in a clinical setting. If this goal of predictive power is met, there will still be a secondary barrier in ensuring that the predictive information is easy to obtain and interpret in clinical practice. Most clinicians would obviously not have the ability to perform advance bioinformatics analyses and computer simulations in their offices! Here statistical tools and machine learning approaches may provide the means to distill insights from detailed bioinformatics analyses and simulations into searchable databases. Such efforts have already been documented for machine learning and data mining applied to diabetes research, as well as other genome-wide association studies [104,105].

## 2. Summary

It has been almost 25 years since KCNH2 was linked to LQTS. Since then we have learned that most LQT2-linked mutations likely decrease IKr by decreasing Kv11.1 channel synthesis or trafficking. The identification of drugs that can improve mutant channel trafficking and increase the open probability could represent a novel therapeutic approach. Perhaps an equally or even more important challenge is harnessing genetic analysis to help identify new LQT2 patients before they suffer a life-threatening arrhythmic event. We expect that recent advances in solving Kv11.1a channel structures, computational simulations, and bio-informatics will improve our ability to distinguish neutral VUS from those that confer a high-risk for LQT2.
